# Multiomics of Colorectal Cancer Organoids Reveals Putative Mediators of Cancer Progression Resulting from SMAD4 Inactivation

**DOI:** 10.1021/acs.jproteome.2c00551

**Published:** 2022-11-30

**Authors:** Jelmer J. Dijkstra, Hannah K. Neikes, Somayeh Rezaeifard, Xuhui Ma, Emile E. Voest, Daniele V. F. Tauriello, Michiel Vermeulen

**Affiliations:** † Department of Molecular Biology, Faculty of Science, Radboud Institute for Molecular Life Sciences (RIMLS), Oncode Institute, Radboud University Nijmegen, Geert Grooteplein 26−28, 6525 GA Nijmegen, The Netherlands; ‡ Department of Cell Biology, Radboud University Medical Center/Radboud Institute for Molecular Life Sciences (RIMLS), Radboud University Nijmegen, Geert Grooteplein 26−28, 6525 GA Nijmegen, The Netherlands; § Department of Molecular Oncology and Immunology, Oncode Institute, The Netherlands Cancer Institute, Antoni van Leeuwenhoek Hospital, 1066 CX Amsterdam, The Netherlands

**Keywords:** colorectal cancer, cancer progression, multiomics, secretomics, SMAD4

## Abstract

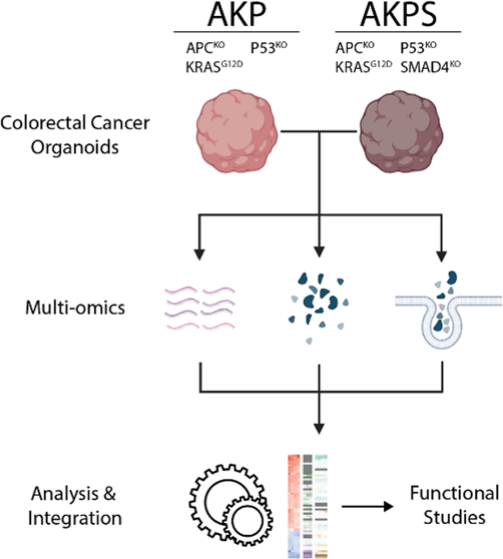

The development of metastasis severely reduces the life expectancy of patients with colorectal cancer (CRC). Although loss of SMAD4 is a key event in CRC progression, the resulting changes in biological processes in advanced disease and metastasis are not fully understood. Here, we applied a multiomics approach to a CRC organoid model that faithfully reflects the metastasis-supporting effects of SMAD4 inactivation. We show that loss of SMAD4 results in decreased differentiation and activation of pro-migratory and cell proliferation processes, which is accompanied by the disruption of several key oncogenic pathways, including the TGFβ, WNT, and VEGF pathways. In addition, SMAD4 inactivation leads to increased secretion of proteins that are known to be involved in a variety of pro-metastatic processes. Finally, we show that one of the factors that is specifically secreted by *SMAD*4-mutant organoids—DKK3—reduces the antitumor effects of natural killer cells (NK cells). Altogether, our data provide new insights into the role of SMAD4 perturbation in advanced CRC.

## Introduction

The transition from healthy gut epithelium to colorectal cancer (CRC) is a multistep process that requires the accumulation of sequential mutations in cancer driver genes.^1^ In most cases, tumor formation starts with an inactivating mutation in the *APC* gene, resulting in constitutive WNT pathway activation that eventually leads to adenoma formation.^2^ Subsequent critical alterations include P53 pathway inactivation and EGFR-pathway activation through mutations in *P*53 and *KRAS*, respectively, further contributing to the progression of adenoma to invasive carcinoma.^3,4^

The next step in CRC progression is the acquisition of metastatic traits. Cancer cells must undergo substantial adaptations, such as increased motility and immune evasion, to be able to migrate to and colonize other parts of the body.^5^*SMAD*4 is a key driver gene for CRC progression, and inactivating mutations that are observed in 5–24.2% of CRC cases are associated with the promotion of metastatic trait development.^6^ The main effects of SMAD4 inactivation on tumor development are thought to occur by regulation of the TGFβ pathway. SMAD4 is a crucial downstream mediator of TGFβ signaling, and its inactivation effectively leads to the inhibition of the canonical TGFβ pathway. This has both direct and indirect effects on the promotion of tumor growth. First, TGFβ signaling has tumor-suppressive roles by controlling cell proliferation, differentiation, and apoptosis within the tumor.^7^ Second, TGFβ desensitization of the tumor allows increased TGFβ levels in the tumor microenvironment without negatively affecting tumor growth by tumor-intrinsic mechanisms.^8^ Increased extracellular TGFβ then promotes the establishment of a pro-metastatic niche through manipulation of the surrounding stromal cells and reduction of the immune response against the tumor, thereby contributing to tumor dissemination.^9,10^ However, SMAD4 inactivation leads to the dysregulation of several TGFβ-independent processes as well, prompting further investigation into hitherto underappreciated functions of SMAD4 in the control of tumor progression.^11−13^

Proteomic and secretomic profiling of CRC samples has been performed before but has mainly focused on the identification of prognostic biomarkers or patient stratification.^14−16^ In addition, most of the studies were performed using CRC cell lines, which often translate poorly to the clinic.^17^ Alternatively, studies performed using patient-derived material accurately reflect the in vivo situation, but attributing dysregulated biological processes to specific mutations is challenging because of the diverse genetic background of the samples. In addition, although the role of SMAD4 in CRC progression is well established, the number of system-wide investigations of the effects of SMAD4 inactivation on CRC progression are limited.^18,19^

The life expectancy of patients drastically falls once a CRC tumor has spread to other parts of the body. Although major improvements in treatment have been made in the last decades, metastatic CRC is still incurable in 80–90% of the cases.^20^ It is therefore essential to gain further insight into the molecular processes that govern the development of metastatic CRC. To this end, we used a human CRC organoid model that faithfully reflects the cancer progression effects of SMAD4 inactivation in advanced CRC. Acquired traits upon SMAD4 inactivation include independence of niche growth factors, development of an invasive carcinoma phenotype, and metastatic potential when introduced in immune-deficient mice.^4,21^ By applying a multiomics strategy, we investigated the effects of SMAD4 inactivation on both intracellular mRNA and protein levels, and on protein secretion dynamics. We show that SMAD4 inactivation in advanced CRC predominantly activates processes involved in the regulation of cell motility and proliferation. In addition, SMAD4 inactivation not only leads to disruption of the TGFβ and BMP pathways but also has significant effects on the activity of other major oncogenic pathways such as WNT, P53, and VEGF. Furthermore, we present a comprehensive list of secreted proteins in CRC organoids that either represent promising targets for intervention or that can be used as biomarkers for metastatic CRC. Finally, we show that the Dickkopf-related protein 3 (DKK3), which is preferentially secreted by metastatic CRC organoids, negatively affects the antitumor properties of natural killer cells (NK cells).

## Materials and Methods

### Recombinant Proteins

Recombinant human DKK3 (1118-DK) and DKK4 (1269-DK) were ordered at R&D systems. DKK3 was dissolved in sterile PBS to a concentration of 250 μg/mL and stored at −80 °C for up to a year. DKK4 was dissolved in sterile 0.1% BSA in PBS and stored at 4°C for up to a month.

Recombinant human IL2 (200-02, PeproTech) was dissolved following the manufacturer’s instructions and stored at −20 °C until further use.

### Organoid Culture

Commercially available Human colon AKP (*APC*^KO^, *KRAS*^G12D^, *P*53^KO^) and AKPS (*APC*^KO^, *KRAS*^G12D^, *P*53^KO^, *SMAD*4^KO^) organoids (Hubrecht Organoid Technology Foundation)^4^ were embedded in a mix of 90% ice-cold RGF BME Type 2 PathClear (Cultrex, R&D Systems) and 10% Dulbecco’s modified Eagle’s medium/F12 (DMEM/F12) (Gibco) and left to polymerize for 15 min at 37 °C. Afterward, organoids were maintained in DMEM/F12 (Gibco), supplemented with 1× Penicillin–Streptomycin (Gibco), 10 mM HEPES (Gibco), 1× Glutamax (Gibco), 1× B27 (Gibco) 1.25 mM *N*-acetylcysteine (Sigma-Aldrich), 50 ng/mL mEGF (Gibco), 10% final volume Noggin conditioned medium, 10 mM nicotinamide (Sigma-Aldrich), 500 nM A83–01 (Cayman Chemical), and 10 mM SB202190 (Cayman Chemical) at 37 °C, 5% CO_2_.

The culture medium was replaced with fresh medium every 2–3 days, and organoids were split 1:4 every 4–5 days, alternating between splitting by mechanical dissociation using a Pasteur pipette and splitting by trypsin-EDTA (Gibco) dissociation.

Organoid collection for proteomic and transcriptomic experiments was performed by incubating the BME-embedded organoids in organoid harvesting solution (Cultrex, R&D Systems) at 10× the BME volume for 45 min at 4 °C while shaking. Organoids were collected by centrifugation at 300*g* for 5 min at 4 °C and washed repeatedly with ice-cold PBS. Organoid pellets were eventually snap-frozen and stored at −80 °C until further processing.

### Cell Culture

NK-92MI cells (ATCC) were cultured in Minimum Essential Medium α without nucleosides (Gibco), supplemented with 12.5% total volume fetal calf serum (HyClone), 12.5% total volume horse serum (Stem Cell Technologies), 2 mM Glutamax (Gibco), 1× penicillin–streptomycin (Gibco), 0.02 mM folic acid (Sigma-Aldrich), 0.2 mM myo-inositol (Sigma-Aldrich), and 0.1 mM 2-mercaptoethanol (Sigma-Aldrich) at 37°C, 5% CO_2_. In addition, 50 U recombinant human IL2 (PeproTech) was supplemented to the culture medium during the first 5 days after starting cell culture.

Subculture of the cells was performed every 2–3 days. Viable cell clusters were collected by centrifugation at 175*g* for 5 min, after which the cells were split in a 1:4 ratio to achieve 2–3 × 10^5^ cells/mL in fresh NK medium.

HT29 cells (ATCC) were cultured in Dulbecco’s modified Eagle’s medium (DMEM) (Gibco) supplemented with 10% fetal bovine serum (Gibco) and 20 mM HEPES at 37 °C, 5% CO_2_.

### Proteomics Sample Preparation

Whole-cell protein extracts were prepared by incubating organoid pellets with SDS lysis buffer (4% SDS, 1 mM DTT, 100 mM Tris-HCl pH 7.5 in ultrapure H_2_O) for 5 min at 95°C. Samples were sonicated until homogeneous using alternating cycles of 30 s on/30 s off on high intensity and spun down at 16 000 for 5 min. The supernatant was transferred to a new tube and protein concentrations were determined using a Pierce BCA protein assay (Thermo Fisher). Afterward, the final DTT concentration was corrected to 100 mM. The organoid lysates were digested with mass spec-grade trypsin (Promega) using filter-aided sample preparation (FASP^22^) and subsequently fractionated using strong anion exchange (SAX^23^). The flow through, pH 11, pH 8, pH 5, and pH 2 fractions were collected. The fractions were desalted and stored on StageTips at 4 °C until measurement by LC-MS/MS.^24^

### Proteomics Mass Spectrometry and Data Analysis

Peptide samples were eluted from StageTips with elution buffer (80% acetonitrile, 0.1% formic acid in ultrapure H_2_O), reduced to 10% of the original volume by vacuum concentration and diluted in 0.1% formic acid to ∼12 μL. The sample (5 μL) was injected and peptides were separated on an Easy-nLC 1000 liquid chromatography system (Thermo Scientific) fitted with a new 30 cm objective emitter of fused silica with an inner diameter of 75 μm packed with C18 beads (ReproSil-Pur, 1.9 μm, 120 A) from Dr. Maish at a flow rate of 250 nL/min using different 214 min gradients of acetonitrile (5–23, 8–27, 9–30, 11–32, and 14–32% for flow through, pH 11, 8, 5, and 2, respectively) followed by washes at 60% followed by 95% acetonitrile for 240 min of total data collection. Data-dependent measurements of the peptides were performed on a Q-Exactive HF-X mass spectrometer (Thermo Scientific). MS1 mass resolution was set to 120.000, the MS1 scan range was 350–1300 *m*/*z*, and MS/MS scan resolution was 15.000. Collision-induced dissociation energy was set at (N)CE 28. Automatic gain control (AGC) was set at 3.00 × 10^6^ and 1.00 × 10^5^ for MS1 and MS/MS, respectively. The AGC intensity threshold for MS/MS was set at 5.00 × 10^4^. Precursors with charge states of 2–5 were selected for fragmentation. For every full scan, the top 20 peptides were selected for fragmentation and dynamic exclusion was set to 30 s with a mass error of 5 ppm.

Protein identification and quantification was done in MaxQuant v1.6.0.1 with default settings, with match-between-runs, iBAQ and label-free quantification enabled. Carbamidomethylation was specified as fixed cysteine modification, and N-terminal acetylation and methionine oxidation were set as variable modifications. The MS/MS spectra were searched against the human Uniprot database including reverse peptide sequences for FDR estimation downloaded in June 2017. Mass tolerance was set at 4.5 and 20 ppm for precursor ion and fragment ions, respectively. FDR was set at 0.01 for both the peptide and protein levels. A minimum of two ratio counts were required for protein quantification

Common contaminants and decoy database hits were removed from the resulting MaxQuant proteinGroups file and alias gene names were replaced with official gene symbols using the Limma package.^25^ If this resulted in duplicate entries, the entry with the highest number of razor + unique peptides was retained. Protein groups were required to have at least two assigned peptides, of which at least one was a unique peptide. Differentially enriched protein analysis was performed using the DEP package.^26^ All protein groups that were detected in at least all but one replicates of at least one condition were considered for downstream analysis. Imputation of missing values was performed using the MinProb method with the default settings. All proteins that showed an adjusted *p*-value < 0.05 and an absolute fold change >1.5 were considered to be differentially expressed.

### Secretomics Sample Preparation

Organoids were left to grow for 5 days after splitting before the start of the experiment. Afterward, the organoid medium was aspirated and organoids were incubated with PBS for 30 min at 37°C to deplete intracellular methionine. Next, the organoids were cultured for 24 h in organoid medium prepared with DMEM/F12 medium without methionine (Gibco), supplemented with 0.1 mM AHA (ThermoScientific) to label nascent proteins. Conditioned medium containing the AHA-labeled secreted proteins was collected and concentrated to 250 μL using 3 kDa centrifugal filters (Amicon) and 1× complete protease inhibitors (CPIs, Roche) were added. The samples were snap-frozen and stored at −80 °C until further processing.

### Enrichment of AHA-Labeled Proteins and On-Bead Digestion

The CuAAC reaction was set up using the Click-iT Protein Enrichment Kit (Invitrogen). In short, 100 μL of alkyne bead slurry was washed with 1 mL ultrapure H_2_O, after which 250 μL of concentrated medium, 250 μL of urea buffer, 500 μL of 2× catalyst solution, and 1× CPIs were added. This was incubated for 16–20 h at room temperature while rotating, after which the beads were washed with 1 mL ultrapure H_2_O. Next, reduction and alkylation of the bound proteins were done by incubating the beads with 10 mM DTT in 500 μL of SDS buffer for 15 min while shaking, followed by incubation with 50 mM iodoacetamide (IAA) in 500 μL of SDS buffer for 30 min while shaking in the dark. The beads were transferred to spin columns and washed with 20 mL of SDS buffer, 20 mL of 8 M urea in 100 mM Tris, pH 8, 20 mL 20% isopropanol, 20 mL 20% acetonitrile, and 5 mL of PBS. The bound proteins were digested by resuspending the beads in 200 μL of freshly prepared digestion buffer (2 M Urea, 100 mM Tris-HCl pH 8, 100 mM DTT) with 0.5 μg of mass spec-grade trypsin (Promega) and overnight incubation at room temperature while shaking. The digest was desalted and concentrated on C18 StageTips without acidification.^24^ Peptide labeling was done by dimethyl labeling,^27^ and StageTips were stored at 4 °C until measurement by LC-MS/MS.

### Secretomics Mass Spectrometry and Data Analysis

Peptide samples were eluted from StageTips with elution buffer (80% acetonitrile, 0.1% formic acid in ultrapure H_2_O), and light and medium labeled samples for the forward and reverse reactions were combined. Next, the samples were reduced to 10% of the original volume by vacuum concentration and diluted in 0.1% formic acid to ∼12 μL. Sample (5 μL) was injected and peptides were separated using an Easy-nLC 1000 liquid chromatography system (Thermo Scientific) with a 44 min acetonitrile gradient (7–30%), followed by washes at 60 and 95% acetonitrile for a total of 60 min data collection. MS settings are described in the Proteomics Mass Spectrometry and Data Analysis section.

Protein identification and quantification was done in MaxQuant v1.5.7.1^28^ with standard settings and requantify enabled. Methionine-to-AHA (−4.98632 Da) and methionine-to-diaminobutanoate (−31.9846 Da) were allowed as variable modifications, in addition to the default N-terminal acetylation and methionine oxidation modifications. Carbamidomethylation was specified as a fixed cysteine modification. Light (+0) and medium (+4) dimethyl labeling on the N-termini and lysine residues was specified under “labels”. The MS/MS spectra were searched against a human Uniprot database downloaded in June 2017. Mass tolerance was set at 4.5 and 20 ppm for precursor ion and fragment ions, respectively. FDR was set at 0.01 for both the peptide and protein levels. Two ratio counts were required for protein quantification.

Maxquant protein groups were filtered as described in the Proteomics Mass Spectrometry and Data Analysis section. All proteins that were detected in both the forward- and reverse-labeled samples of both biological replicates were considered for downstream analysis. The forward and reverse ratios of the two experiments were averaged, and this was used as relative expression values. All proteins with a mean absolute fold change >2 in both the forward- and reverse-labeled experiment were considered to be differentially secreted.

### RNA-Sequencing

RNA was extracted from snap-frozen organoid pellets using the RNeasy RNA extraction kit (Qiagen) with DNaseI treatment. A total of 1 μg of RNA per replicate was used as input to generate RNA-seq libraries with the KAPA Stranded RNA-Seq Kit with RiboErase (HMR), following the manufacturer’s instructions with the following adjustments. Fragmentation of RNA was performed for 6.5 min at 94 °C for a desired library insert size of 200–300 bp, and at the end of the library preparation, libraries were subjected to a 0.8× clean-up, followed by a 1× clean-up. Library concentrations were measured using the KAPA Library Quantification Kit (KAPA Biosystems), and library size was determined using the BioAnalyzer High Sensitivity DNA Kit (Agilent). Sequencing libraries were paired-end sequenced with an Illumina NextSeq500 to a read length of 38 bp.

### RNA-Sequencing Data Analysis

Sequenced reads were aligned to the human hg38 genome with HISAT2.^29^ Duplicate reads were removed with PICARD (http://broadinstitute.github.io/picard) and count tables for downstream analyses were generated with HTSeq.^30^

Differential gene expression analysis was performed using the R DESEQ2 package.^31^ Pre-filtering was performed by removing all genes with <10 reads. All genes with an adjusted *p*-value <0.05 and absolute FC > 1.5 were considered to be differentially expressed. Regularized log transformation was applied for visualization purposes.

### Integration of Omics Data and Data Visualization

The different data sets were matched based on their respective associated gene symbol and mapping of transcript/protein IDs to gene symbols was done using BioMart.^32^ Heatmaps were created using ComplexHeatmap^33^ and other data visualizations were created with ggplot2.^34^ Schematic figures were made using Biorender.

### Gene Set Enrichment, Over-Representation, and Pathway Analysis

Gene set enrichment analysis^35^ was performed using the clusterProfiler package.^36^ The output of DESEQ2 ranked on fold change was used as gene list. All gene sets belonging to the Gene Ontology: Biological Pathway category C5 collection were included. The results were ranked in increasing order based on adjusted *p*-value.

Over-representation analysis was performed with DAVID 2021.^37^ All secreted proteins that were found in all replicates were used as input list and tested for over-representation in the gene sets belonging to the Gene Ontology: Biological pathway category C5 and Gene Ontology: Cellular compartment category C5 collections. The results were ranked in increasing order based on FDR.

Pathway analysis was performed using PROGENy.^38^ Transcriptomic fold changes resulting from DESEQ2 analysis were used as input.

### TCGA Data Analysis

Transcriptome and mutation data for the TCGA-COAD cohort was downloaded using the R “TCGA-biolinks” package.^39^ For the mutations data, all mutations classified as ‘Frame_Shift_Del’, ‘Frame_Shift_Ins’, ‘In_Frame_Del’, ‘In_Frame_Ins’, ‘Missense_Mutation’, ‘Nonsense_Mutation’, ‘Splice_Site’, and ‘Translation_Start_Site’ were considered. B/C samples were removed from the gene expression data so that there was one sample per patient. All patients with both transcriptome and mutation data were considered for survival analysis. Kaplan–Meier curves for survival analysis were created using the R “survminer” and “survival” packages. For all survival plots made, the “high” group consisted of patients with gene expression in the top 50%, the “low” group consisted of patients with gene expression in the bottom 50%.

### Generation of Tumor Organoids Reactive PBMC

Tumor-reactive patient T cells were generated by coculturing PBMCs and autologous tumor organoids as described previously.^40,41^ Briefly, patient tumor organoids were isolated from Geltrex (Gibco) 48 h prior to coculture and stimulated with 200 ng/mL IFNγ (PeproTech, 300-02) for 24 h prior to coculture. On the day of coculture, tumor organoids were dissociated into single-cell suspension using TripLE Express (Gibco). Tumor organoid cells (5 × 10^3^) mixed with 1 × 10^5^ patient PBMCs (1:20 tumor cell/PBMC ratio) were seeded in each well of a U-bottom 96-well plate precoated with 5 μg/mL anti-CD28 antibody (eBioscience, CD28.2, 16-0289-81) and left to attach overnight. The coculture medium consisted of RPMI 1640 (Gibco) supplemented with 2 mM Ultraglutamine I (Lonza), penicillin/streptomycin (Gibco), 10% human AB serum (Sigma-Aldrich), 150 U/mL rh-IL-2 (Proleukin, Novartis), and 20 μg/mL anti-PD1 blocking antibody (Merus, The Netherlands). Medium, IL-2, and anti-PD1 were refreshed every 2–3 days. PBMCs were harvested and restimulated every 7 days by replating with fresh tumor organoid cells. After 2 weeks of coculture, PBMCs were harvested and used for downstream analysis or cryopreserved for later use.

### Tumor Organoids Killing Assay

Tumor-reactive PBMC were thawed in prewarmed T-cell medium and incubated for 15 min with 25 U/mL Benzonase. After washing, the cells were resuspended at 2–3 × 10^6^ cells/mL in T-cell medium and cultured overnight at 37 °C.

Organoids were isolated from Geltrex 48 h prior to coculture and stimulated with 200 ng/mL IFNγ for 24 h prior to coculture. On the day of coculture, part of the organoids was dissociated into single cell that was used for counting and the rest of the fully formed organoids were used for the experiment. The number of “single-cell equivalents” tumor cells of organoids was calculated. Next, 1 × 10^5^/mL single-cell equivalents organoids and 5 × 10^5^/mL tumor-reactive T cells were resuspended in the T-cell medium, respectively. Anti-CD28 coated plates were washed twice with PBS and 1 × 10^4^ organoids were seeded with 5 × 10^4^ T cells or without T cells. The cocultures were treated by DKK3 at the doses of 0, 1.25, 5, and 20 μg/mL or by DKK4 at the doses of 0, 0.25, 1, and 4 μg/mL for 72 h in triplicate.

To quantify T-cell-mediated killing, the cells were harvested at the end of culture, washed twice with 200 μL of PBS, and dissociated to single cells. Counting beads (5 μL) were added per well. After washing twice with PBS, the cells were stained with anti-CD3-FITC antibody (Biolegend, cat. no. 344804), anti-EpCAM-PE antibody (Biolegend, cat. no. 324206), and near-IR viability dye (Invitrogen, cat. no. L10119). Plates were incubated for 30 min at 4°C in the dark and then washed twice with 200 μL of FACS buffer (0.5M EDTA and 0.1% BSA in PBS). The cells were resuspended in 50 μL of FACS buffer and then recorded on a flow cytometer (BD, Fortessa).

### Natural Killer Cell Killing Assay

NK-92MI cells (6 × 10^5^ cells/mL) were treated with DKK3 (0–20 μg/mL) and DKK4 (0–4 μg/mL) for 24 h. HT29 cells were washed with PBS and detached using Trypsin (Gibco), then seeded in a 96-well plate (1.7 × 10^4^ cells/well) for the experiment. HT29 cells were left to attach to the bottom of the wells for 4 h at 37 °C, 5% CO_2_, after which the DKK-treated NK-92MI cells were added to HT29 cells (NK92-MI/HT29-ratio = 2). After 24 h coculture, the cell supernatants were discarded and HT29 cells were washed with PBS and detached using Trypsin. Detached cells were then suspended in 100 μL of 10% FBS-DMEM and recorded by a MACSQuant flow cytometer (Miltenyi Biotec) after propidium iodide (PI) addition.

### Data Availability

Next-generation sequencing data have been deposited at Gene Expression Omnibus (GEO) with accession code GSE114113. The mass spectrometry data have been deposited at the ProteomeXchange Consortium via the PRIDE partner repository with the data set identifier PXD036441. Additional data files showing detected features and statistics for transcriptomic, proteomic, and secretomic experiments are provided as Supporting Data Files.

## Results

### CRC Organoids Provide a Powerful Tool to Study Metastasis on a Molecular Level

**Figure 1:**
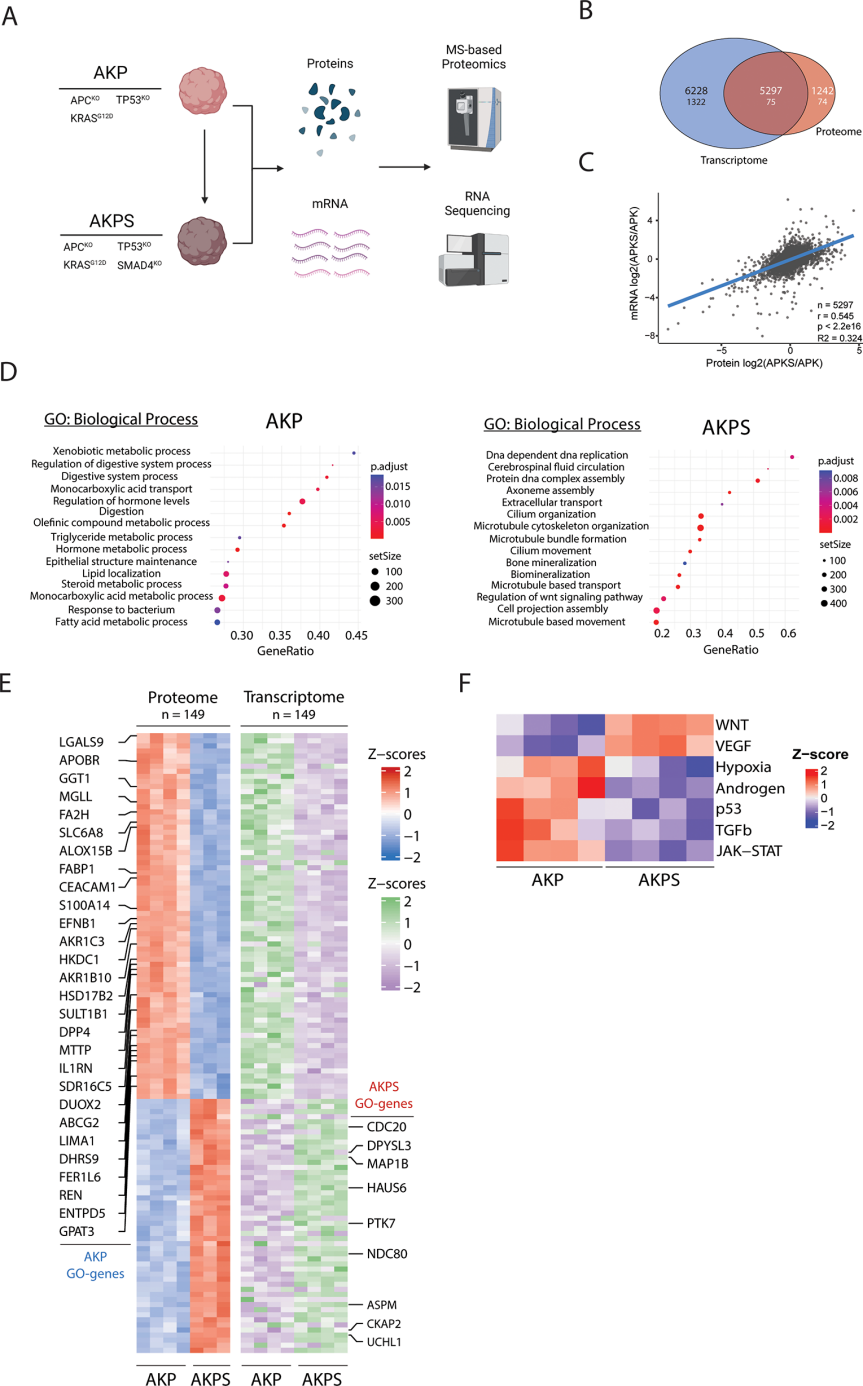
Transcriptomic and proteomic analysis of AKP and AKPS organoids. (A) Schematic overview of the used organoid lines and experimental procedures. (B) Venn diagram of detected and significant (FDR < 0.05 and FC > 2) transcriptomic and proteomic features. The upper number shows detected features, and the lower number shows significantly changing features. (C) Linear regression of relative transcriptomic and proteomic expression levels of AKP vs AKPS. *n* = 5297, *r* = 0.545, *R*^2^ = 0.324, *p* < 2.2 × 10^–16^. (D) Bubble plots showing the top 15 activated biological processes ranked on increasing FDR based on GSEA. All reported biological processes are over-represented with an FDR < 0.01. The left panel shows AKP-specific processes, and the right panel shows AKPS-specific processes. (E) Row-matched heatmaps showing the relative changes in protein and mRNA expression of the 149 significantly changing proteins shown in (B) (FDR < 0.05; FC > 1.5). Highlighted rows show significantly changing proteins that are associated with the biological processes in (D). (F) Heatmap showing significantly (FDR < 0.05) the relative activation of PROGENy pathways in AKP and AKPS organoids.

To study the role of SMAD4 in CRC progression toward metastatic competency, we adopted a CRC tumor progression organoid (TPO) model (Figure 1A).^4^ This model was developed to mimic the adenoma-to-carcinoma progression in CRC by the sequential introduction of mutations in four frequently mutated oncogenes (*APC* (A), *KRAS* (K), *P*53 (P), and *SMAD*4 (S)) by CRISPR/Cas9 in human colon organoids derived from human-derived healthy colon epithelium, providing an excellent isogenic organoid model to study the role of SMAD4 inactivation on cancer progression in advanced CRC on a molecular level.

We isolated RNA and proteins from AKP and AKPS organoids and investigated transcriptomic and proteomic dynamics by bulk RNA-sequencing and mass spectrometry-based proteomics, respectively (Figure 1A). This resulted in the identification and quantification of 11526 protein-coding transcripts and 6539 protein groups (Data S1 and S2). 1399 transcripts and 149 protein groups were dynamically regulated between the AKP and AKPS organoids, of which 75 features were dynamically regulated at both levels (Figure 1B). Correlation analysis of all 5927 features that were found in both data sets showed a moderate to strong correlation between the transcriptome and proteome (*r* = 0.545) (Figure 1C).

The transcriptomic data were used to identify the biological processes that are affected by SMAD4 deactivation (Figure 1D). AKP organoids mainly showed increased activity of metabolic processes, whereas activated processes in AKPS organoids are mainly involved in cell proliferation and movement. This is in line with previous studies showing that AKPS organoids show a loss of epithelial cell identity and have increased proliferation.^42,43^ Further investigation into the molecular regulators of the identified biological processes revealed multiple differentially expressed proteins and transcripts that have a potential role in cancer progression (Figure 1E). For example, cell division cycle protein 20 homolog (CDC20) is an essential co-factor for the anaphase-promoting complex (APC/C), which is an important regulator of mitosis. Overexpression of CDC20 predicts a poor prognosis for CRC patients, but the precise molecular processes in which CDC20 is involved in CRC are largely unknown.^44^

Finally, transcriptomic data were used to investigate differences in the activity of a selected group of important oncogenic signaling pathways were investigated using PROGENy^38^ (Figure 1F). Reassuringly, SMAD4 inactivation led to the downregulation of the TGFβ pathway in AKPS organoids. To our surprise, however, several other pathways showed significant differences in activity between AKP and AKPS organoids. Of these, the WNT and P53 pathways were the most notable, as both pathways are disrupted in AKP and AKPS organoids by inactivating mutations in *APC* and *P*53, respectively. This suggests extensive crosstalk between major signaling pathways that is partly mediated by SMAD4. Indeed, it was demonstrated that SMAD4 inactivation has synergistic effects on the WNT and P53 pathways, warranting further investigation of the effects SMAD4 loss on other pathways, such as the VEGF pathway.^13,45^

### Secretomics Reveals Many Putative Mediators of CRC Metastasis

Dysregulated protein secretion is commonly observed in cancer and plays an important role in the development of metastasis via autocrine stimulation of pro-metastatic processes and paracrine modulation of the tumor microenvironment.^76,77^ Therefore, we set out to characterize the differences in protein secretion between AKP and AKPS organoids.

**Figure 2:**
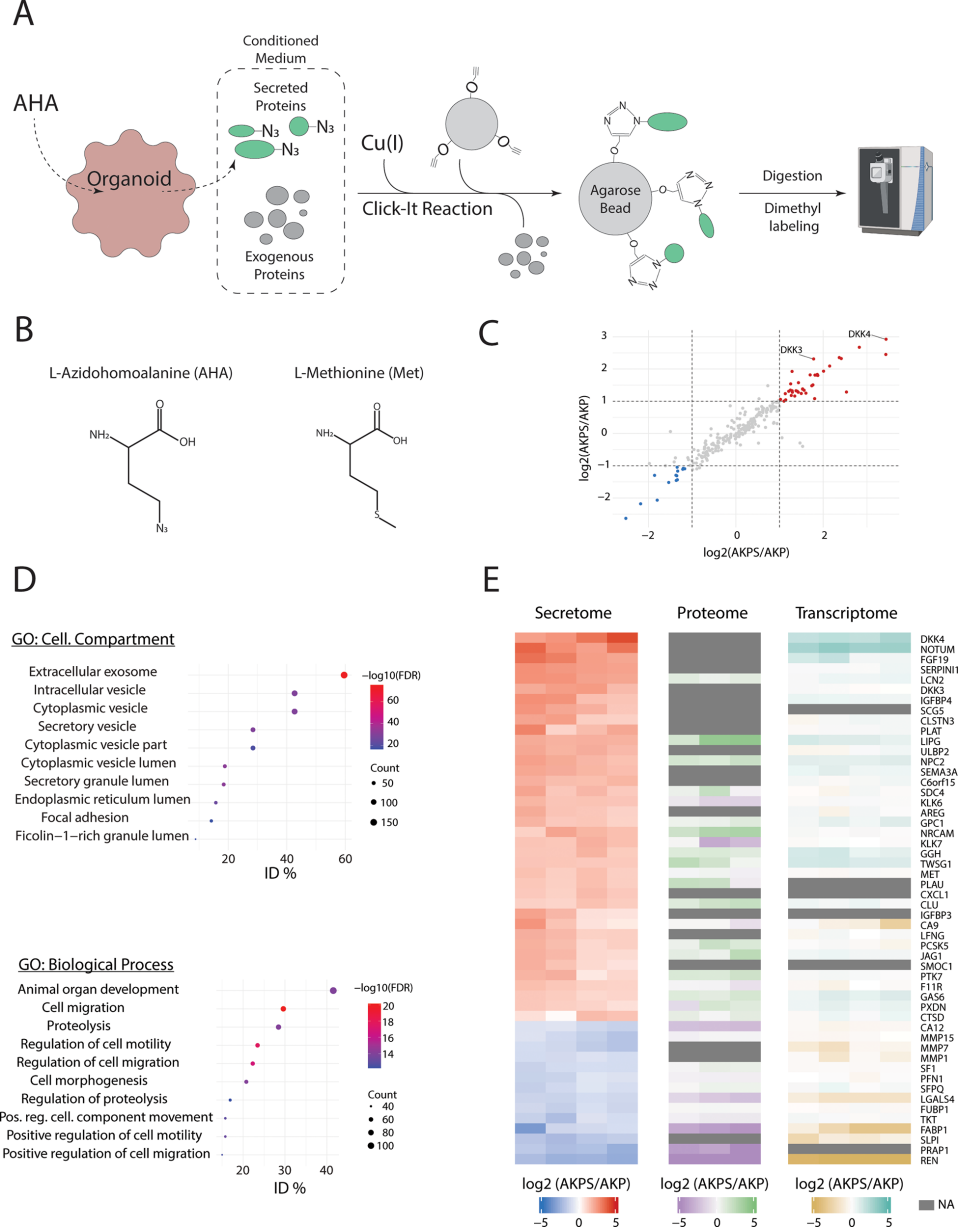
Secretomic analysis of AKP and AKPS. (A) Schematic overview of the application of BONCAT in secretomics. (B) Structure formulas of methionine (Met) and azidohomoalanine (AHA). (C) Dot plot showing changes in relative protein secretion levels between AKP and AKPS organoids. Proteins with an absolute mean FC > 2 were considered to be significantly differentially secreted. (D) Bubble plots showing the top 10 significantly (FDR < 0.01) enriched biological processes and cellular compartments ranked on increasing FDR based on ORA using all detected proteins. All reported biological processes and cellular compartments are over-represented with an FDR < 0.01. (E) Row-matched heatmaps showing the relative expression levels of secreted proteins (left), intracellular proteins (middle), and mRNA transcripts (right). All secreted proteins with a fold change >2 (colored dots in (C)) are shown.

A known challenge in the detection of secreted proteins in conditioned medium (CM) is to distinguish low-abundant cell-derived proteins from exogenous cell culture medium proteins. To overcome this issue, we combined bio-orthogonal non-natural amino acid tagging (BONCAT) with MS-based proteomics.^78^ BONCAT relies on the incorporation of tagged amino acid analogues in nascent proteins. In this case, methionine (Met) is replaced with the azide-bearing analogue azidohomoalanine (AHA), which facilitates the isolation of secreted proteins from CM. AKP and AKPS organoids are treated with AHA for 24 h, after which the CM is collected. The AHA-tagged proteins are then isolated by covalently binding the proteins to alkyne-containing agarose beads using a Click-reaction. After stringent washing, the secreted proteins are analyzed by MS (Figure 2A,B).

This resulted in the detection of a total of 260 secreted proteins, of which 14 proteins were considered to be preferentially secreted by AKP organoids and 37 proteins by AKPS organoids (Figure 2C and Data S3). We then used all 260 detected proteins as input for gene ontology (GO) over-representation analysis (ORA) to identify the cellular compartments and biological processes that are associated with the secreted proteins (Figure 2D). Reassuringly, the majority of the cellular compartments hereby identified were involved in protein secretion, indicating that potential contamination of intracellular proteins was minimal. This is confirmed by inspecting the Uniprot keywords associated with the identified proteins, showing that keywords associated with secretion and cell membrane localization are among the most frequently observed keywords (Figure S1). Furthermore, the secreted proteins are associated with biological processes that are highly relevant for metastasis, such as proteolysis of the extracellular matrix and regulation of cell motility, underscoring the role of SMAD4 inactivation in the promotion of metastasis. The role of several proteins that are preferentially secreted by AKPS organoids in metastasis formation is highlighted in Table 1.

**Table 1:** Overview of Selected Proteins That Are Preferentially Secreted by AKPS Organoids and Their Roles in Metastatic Processes

name	gene	protein	canonical function	role in CRC progression	references
Dickkopf-related protein 3	DKK3	DKK3	negatively regulates Wnt signaling by inhibiting LRP5/6 interaction with WNT	promotes pro-oncogenic properties CAFs; reduces immune cell activity; modulates cancer cell proliferation, migration, and invasiveness	(46−50)
Dickkopf-related protein 4	DKK4	DKK4	negatively regulates Wnt signaling by inhibiting LRP5/6 interaction with WNT	promotes tumor invasion and angiogenesis; reduces immune cell infiltration; increases drug resistance	(51−54)
palmitoleoyl-protein carboxylesterase NOTUM	NOTUM	NOTUM	negatively regulates Wnt signaling by depalmitoleoylation	promotes clonal fixation; promotes tumor growth and metastasis	(55−57)
fibroblast growth factor 19	FGF19	FGF19	suppresses bile acid biosynthesis through CYP7A1 downregulation	promotes tumor growth; enhances EMT	(58−60)
UL16-binding protein 2	ULBP2	ULBP2	binds and activates the KLRK1/NKG2D receptor, mediating natural killer cell cytotoxicity	reduces NKC infiltration and activity	(61, 62)
neuroserpin	SERPINI1	SERPINI1	serine protease inhibitor that inhibits plasminogen activators and plasmin but not thrombin	enhances EMT; reduces cell adhesion	(63, 64)
neutrophil gelatinase-associated lipocalin	LCN2	NGAL	iron-trafficking protein involved in multiple processes such as apoptosis, innate immunity, and renal development	promotes tumor progression; confers 5-FU resistance; regulates metastasis	(65−70)
kallikrein-6	KLK6	KLK6	serine protease	promotes cell migration and invasion	(71−73)
insulin-like growth factor-binding protein 4	IGFBP4	IGFBP4	binds to IGFs, thereby prolonging their half-lives	increases apoptosis; reduces angiogenesis; reduces cell proliferation and migration	(74, 75)

Finally, we compared the dynamics in protein secretion with the matched intracellular protein and transcript levels (Figure 2E). Although in general a similar trend can be observed across the three levels of regulation, there are notable exceptions. Most notably, several secreted proteins (e.g., DKK4, NOTUM, FGF19) could not be detected intracellularly, even though LFQ intensity values were determined for more than 6000 intracellular proteins. Furthermore, several secreted proteins showed opposite dynamics relative to intracellular protein and mRNA levels (e.g., KLK6, KLK7, CA9). Protein secretion dynamics are more relevant to predict effects on the establishment of a metastatic niche, as they are the end-point of the signaling cascade. This underscores the importance of investigating not only intracellular dynamics but also extracellular proteins dynamics, as many putative regulators of metastasis may otherwise be overlooked.

### DKK3 Reduces the Ability of NK Cells to Kill CRC Cells

**Figure 3:**
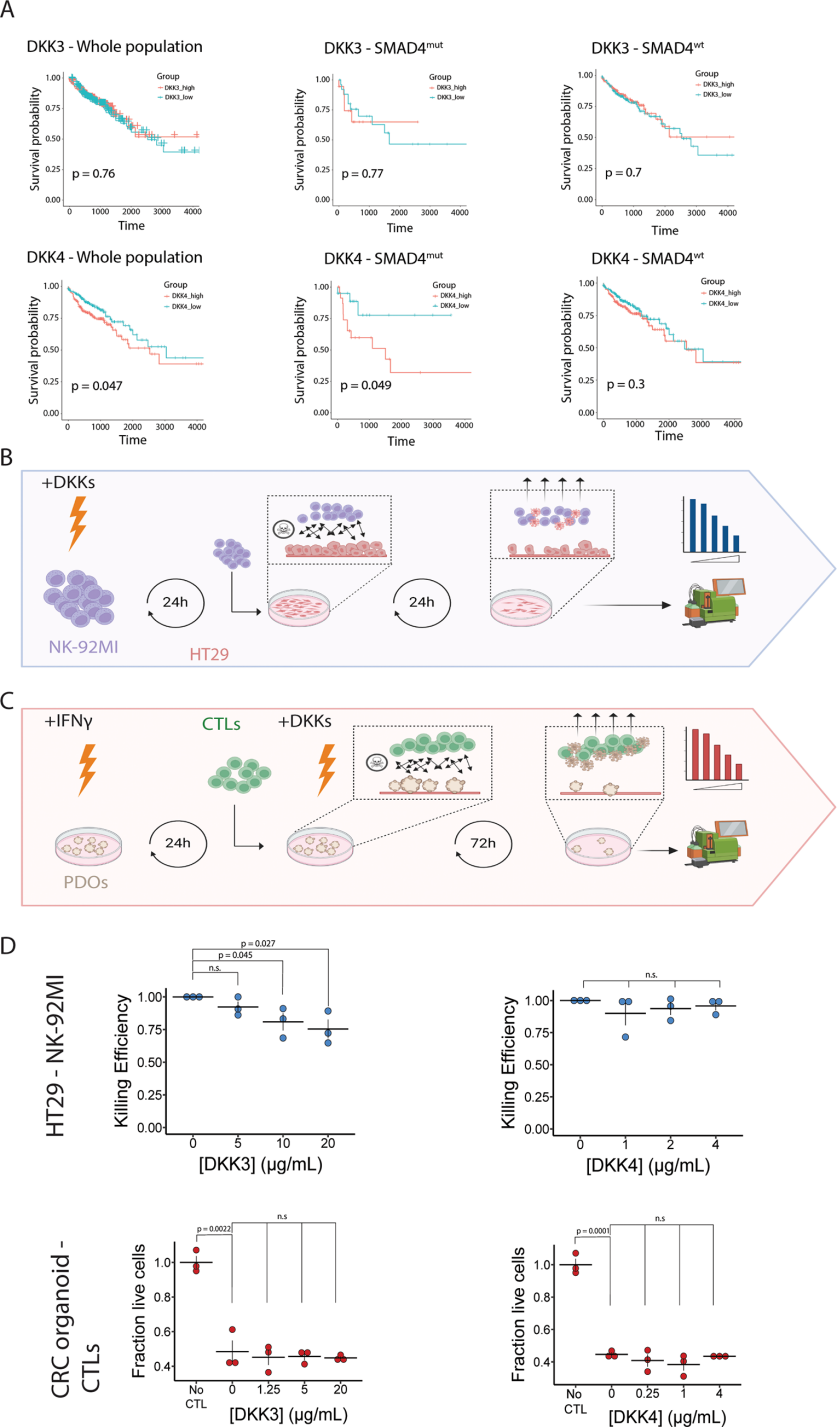
DKK3 reduces the killing capacity of NK cells for CRC cells in vitro. (A) Kaplan–Meier plots showing the effect of DKK3 (top) and DKK4 (bottom) expression on survival probability in the TCGA-COAD cohort. Left panels include the whole cohort, middle panels show patients with mutations in SMAD4, and right panels show patients without SMAD4 mutations. (B) Schematic overview of killing assays using CRC cells and NK cells. NK-92MI cells are incubated for 24h with recombinant DKK-proteins after which they are co-cultured with HT29 cells. After 24h of coculture, non-adherent cells are removed and adherent cells are dissociated and counted by flow cytometry (C) Schematic overview of killing assays using patient-derived CRC tumor organoids and matched CTLs. Patient derived organoids (PDOs) are incubated with IFNγ for 24h after which they are cocultured with CTLs in combination with recombinant DKK proteins. After 72h, non-adherent cells are removed and adherent cells are dissociated and measured by flow cytometry (D) Dot plots showing the effect of DKK3 and DKK4 on the antitumor capacity of NK cells (top) and CTLs (bottom). No CTL indicates the control condition, where no CTLs are added to the tumor organoids; *n* = 3 independent replicates; error bars represent SEM. n.s: not significant.

We used the list of secreted proteins to identify targets for follow-up (Figure 2E). Notably, two proteins of the Dickkopf (DKK) family of proteins (DKK3 and DKK4) were among the top ranked proteins that are preferentially secreted by AKPS organoids. The DKK family consists of four closely related proteins that are mainly associated with the regulation of the WNT signaling pathway. Both AKP and AKPS organoids are insensitive to the regulation of the WNT/β-catenin pathway by extracellular ligands as a result of APC inactivation,^79^ suggesting that increased DKK secretion is responsible for the regulation of other processes as well. We first mined the TCGA-COAD data set to investigate the effect of high DKK3 and DKK4 expression on survival probability in a cohort of colon cancer patients (Figure 3A). High DKK3 expression does not have an effect on overall survival, irrespective of SMAD4 mutations status. However, high DKK4 expression leads to a worse prognosis in the complete population, and the detrimental effects of DKK4 are exacerbated by the presence of SMAD4 mutations, suggesting a synergistic effect of SMAD4 inactivation and DKK4 on patient survival.

Several members of the DKK family are involved in the regulation of immune activity. As immune evasion is a critical step for metastasis, we reasoned that AKPS organoids might preferentially secrete DKK proteins to negatively regulate the antitumor activity of immune cells, thereby contributing to a worse prognosis for patients. We therefore decided to investigate whether DKK3 and DKK4 inhibit the ability of NK cells and cytotoxic T-lymphocytes (CTLs) to kill CRC cells.

To test the effect of DKK proteins on immune cell activity, we set up a coculture killing assay for NK-92MI cells with the HT29 CRC cell line (Figure 3B) and patient-derived CTLs with matched patient-derived CRC tumor organoids (Figure 3C). The immune cells were cocultured with CRC cells in the presence and absence of DKK3 or DKK4, after which the killing efficiency of NK cells was established by flow cytometry.

To our surprise, out of the four combinations tested, only DKK3 showed a dose-dependent effect on the killing capacity of NK cells (Figure 3D). This suggests that increased secretion of DKK3 by AKPS organoids reduces NK-cell-mediated tumor killing, which may contribute to the metastatic potential of SMAD4-knockout organoids. In the absence of protein secretion data, mRNA expression was used for patient stratification. However, DKK3 mRNA expression and DKK3 secretion correlate poorly in the used organoid model (Figure 2E), offering a potential explanation for the apparent difference in effect of DKK3 on patient survival, and its effect on NK cell activity. As for DKK4, it could be that it exerts its effects in an autocrine manner, which was not tested in this study.

## Discussion

The acquisition of metastatic traits defines the final stage of CRC progression. SMAD4 inactivation can drive the development of metastatic traits in a subset of CRC cases, but a system-wide study of the effects of SMAD4 inactivation on the molecular mechanisms and related biological processes that contribute to CRC progression was not performed yet. In this study, we applied a multiomics approach to study the molecular consequences of SMAD4 inactivation in advanced CRC at the transcriptome, proteome, and secretome levels. Based on the data presented in this study, we propose three SMAD4-mediated processes that may contribute to CRC progression toward metastatic disease, although we cannot exclude the possibility that loss of SMAD4 influences other pro-metastatic processes as well.

First, loss of SMAD4 leads to the activation of noncanonical WNT and TGFβ signaling pathways. To our surprise, pathway analysis showed that SMAD4 inactivation leads to activation of the WNT pathway, despite the fact that constitutive β-catenin mediated WNT signaling is active in both AKP and AKPS organoids as a result of APC knockout. We reason that this could be the result of alternative WNT signaling pathway activation, e.g., the planar cell polarity (PCP) pathway, which is a β-catenin-independent WNT signaling pathway that is associated with increased cell motility, metastasis, and cell proliferation.^80,81^ This is supported by the upregulation of inactive tyrosine-protein kinase 7 (PTK7) at the transcriptomic, proteomic, and secretomic levels in AKPS organoids. PTK7 acts as both an inhibitor of canonical WNT signaling and as activator of WNT-PCP signaling, and its upregulation results in increased cell motility, migration, and metastasis.^82−84^ In addition, PCP pathway activation leads to activation of YAP/TAZ signaling, which results in a pro-migratory metastatic phenotype that is crucial for liver colonization by CRC cells.^85,86^ Concomitantly, YAP/TAZ activation leads to the increased secretion of WNT inhibitors by cancer cells. Our secretomics data show increased secretion of several inhibitors of canonical WNT signaling, such as DKK3, DKK4, and NOTUM, further supporting the role of SMAD4 as a mediator of PCP and YAP/TAZ signaling. In addition, SMAD4 inactivation can shift the balance from canonical to noncanonical TGFβ signaling, thereby favoring metastatic development through Par6 signaling and cell proliferation through activation of MAPK signaling.^87−89^

Second, loss of SMAD4 induces cancer cell dedifferentiation and promotes cancer stem cell (CSC) development. We show that SMAD4 inactivation leads to the downregulation of metabolic processes that are associated with differentiated gut epithelium. Loss of differentiation and the acquisition of CSCs are crucial for metastasis, and AKPS organoids show increased numbers of CSCs.^90,91^ TGFβ and BMP signaling have opposite effects on the maintenance of cellular identity in CRC, with BMP4 inhibiting CSC formation, whereas TGFβ signaling promotes EMT and CSC dedifferentiation.^92,93^ However, both processes are not directly regulated by SMAD4, suggesting a dependency on other signaling pathways or the activation of noncanonical signaling pathways.^94−96^ Indeed, it was shown that SMAD4 inactivation leads to enhancement of WNT/β-catenin pathway, thereby driving CRC dedifferentiation.^13,97,98^ Another potential indirect mechanism by which SMAD4 inhibition promotes CSC formation is through the disruption of hormone metabolism, most notably in the synthesis of steroid hormones. We previously showed that the hormone-activated nuclear receptor (NR) Retinoid X Receptor (RXR) is important for the maintenance of cell identity in CRC.^42^ Other NRs, such as the Farnesoid X Receptor (FXR) and the thyroid hormone receptor (TR), are involved in the control of CSC proliferation and differentiation as well.^99,100^ Together, this suggests a role for SMAD4 in the maintenance of cellular identity by controlling NR activity through the regulation of ligand availability.

Finally, SMAD4 inactivation leads to increased expression of pro-tumorigenic factors. Secretomic analysis revealed the increased secretion of multiple proteins that promote metastasis through autocrine mechanisms, such as induction of EMT and cell proliferation, as well as through paracrine mechanisms, including the promotion of angiogenesis and immune cell modulation (Table 1). However, as many detected proteins have an as yet unknown role in CRC progression, these proteins represent putative new targets for follow-up studies. We focused on the role of two of the top ranked proteins that are preferentially secreted by AKPS organoids: DKK3 and DKK4. The DKK protein family is traditionally known to regulate WNT signaling, but all four members are associated with the modulation of the antitumor immune response as well (Table 1).^101−103^ We found that DKK3, but not DKK4, was able to negatively affect the killing capacity of NK cells for CRC cells, but that neither was able to decrease the activity of CTLs in a patient-derived colon cancer model. Both DKK3 and DKK4 have been reported to dampen the antitumor response in CTLs, but not in the context of CRC, providing a possible explanation for the absence of an effect on CTL response. In addition, DKK4 activity is dependent on extracellular proteolysis.^104^ As we used full-length recombinant DKK4, it is possible that this was not converted to the active form. Furthermore, although the tested CTLs are reactive to the tumor organoids, as shown by the strong decrease in live cells after the addition of CTLs (no CTL vs 0 conditions), no effects of the DKKs on their killing capacity could be observed. There are several potential explanations for the discrepancy in the effect of DKK3 on the killing capacity of immune cells in the HT29 and PDO experiments. First, NK cells were used in the HT29 experiments, whereas CTLs were used in the organoid experiment, suggesting that NK cells, but not CTLs, are affected by DKK3. Second, the HT29 cell line and used organoid lines have likely different genetic backgrounds. It is possible that the used organoid line acquired mutations that render it resistant to DKK3-induced changes in CTL activity. Finally, the HT29 model is a less complex model than the organoid model, which e.g., reflects in vivo tumor heterogeneity better.

In summary, we show that SMAD4 inactivation in advanced CRC leads to molecular changes at the transcriptome, proteome, and secretome levels. We propose three potential mechanisms by which SMAD4 inactivation may promote metastasis: activation of alternative WNT and TGFβ signaling; loss of differentiation and development of CSCs; and increased expression of pro-tumorigenic proteins. This is, however, far from exhaustive and our data provide many new promising avenues for further investigation into the pro-metastatic properties of SMAD4 inactivation in CRC.

## Supporting Information Available

Barplot showing Uniprot keywords associated with proteins identified in secretome experiments (Figure S1) (PDF)
Raw RNA-sequencing read counts and results of DGE (Data S1) (XLSX)
MaxQuant proteinGroup results, an overview of LFQ intensities and the result of DPE (Data S2) (XLSX)
MaxQuant proteinGroup results, ratios of labeled proteins and the result of DPE (Data S3) (XLSX)
