# Supplementary material for: Multiomics of Colorectal Cancer Organoids Reveals Putative Mediators of Cancer Progression Resulting from SMAD4 Inactivation
Source: J Proteome Res. 2022 Nov 30;22(1):138–51. doi: 10.1021/acs.jproteome.2c00551 (PMC9830641; doi:10.1021/acs.jproteome.2c00551)
Supplement: Supplementary file 1 — pr2c00551_si_002.pdf [file pr2c00551_si_002.pdf]

Supporting Figures.

Figure S1

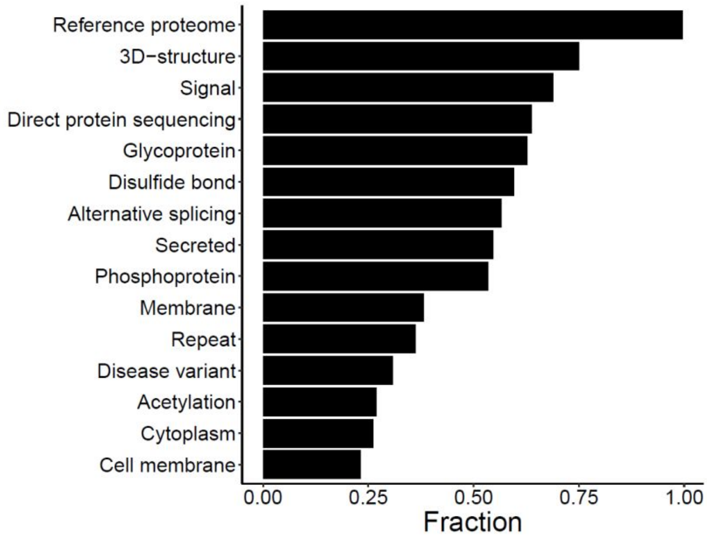

Figure S1. Barplot showing the frequency of Uniprot keywords that are associated with identified secreted proteins.
